# Resistance evolution under potentiated sulphonamide pressure in *Escherichia coli*

**DOI:** 10.3389/fvets.2025.1697872

**Published:** 2025-11-26

**Authors:** Ádám Kerek, Bence Török, Ábel Szabó, Levente Laczkó, Gábor Kardos, Krisztián Bányai, Eszter Kaszab, Krisztina Bali, Ákos Jerzsele

**Affiliations:** 1 Department of Pharmacology and Toxicology, University of Veterinary Medicine Budapest, Budapest, Hungary; 2 National Laboratory of Infectious Animal Diseases, Antimicrobial Resistance, Veterinary Public Health and Food Chain Safety, University of Veterinary Medicine Budapest, Budapest, Hungary; 3 One Health Institute, University of Debrecen, Debrecen, Hungary; 4 HUN-REN–UD Conservation Biology Research Group, Debrecen, Hungary; 5 Department of Bioinformatics, University of Debrecen, Debrecen, Hungary; 6 National Public Health Center, Budapest, Hungary; 7 Department of Gerontology, Faculty of Health Sciences, University of Debrecen, Nyíregyháza, Hungary; 8 Department of Medical Biology, Medical School, University of Pécs, Pécs, Hungary; 9 HUN-REN Veterinary Medical Research Institute, Budapest, Hungary; 10 Department of Microbiology and Infectious Diseases, University of Veterinary Medicine, Budapest, Hungary

**Keywords:** microevolution, co-selection, MEGA-plate, *Escherichia coli*, potentiated sulphonamide, NGS, AMR, resistance development

## Abstract

**Introduction:**

Antimicrobial resistance (AMR) poses an escalating global health threat. Potentiated sulphonamides are widely used in both veterinary and human medicine. This study aimed to investigate the *in vitro* adaptation of *Escherichia coli* strains to increasing concentrations of potentiated sulphonamides, focusing on co-selection and the genetic mechanisms of resistance.

**Methods:**

The MEGA-plate evolutionary model was used to expose *E. coli* ATCC 25922 to increased concentrations (0 × to 1000×) of a potentiated sulphonamide. Clones isolated from different concentration zones were analyzed for phenotypic resistance via minimum inhibitory concentration (MIC) testing and genotypically through next-generation sequencing.

**Results:**

In strains adapted to 1000 × potentiated sulphonamide, MIC values significantly increased for most tested antibiotics. Mutations were identified in key folate pathway genes (*folP*, *folA*), as well as in efflux pump regulator genes (*emrR*, *marR*, *acrR*, *mdtM*). These genetic changes indicated activation of multiple multidrug efflux systems, including *acrAB-tolC*, *emrAB-tolC*, and *mdtEF-tolC*. Mutations were also detected in genes associated with SOS response regulation (*recN*, *recQ*, *uvrB*), suggesting stress-induced genetic adaptation. *In vitro* microevolutionary adaptation to potentiated sulphonamide exposure induced broad genetic changes in *E. coli*, potentially driving cross-resistance through co-selection. The MEGA-plate method proved to be a robust tool for tracking resistance development and dissecting complex resistance mechanisms.

**Discussion:**

These findings underscore the need for cautious use of combination antimicrobials, as they may elicit pleiotropic resistance responses beyond their intended targets.

## Graphical abstract

**Graphical Abstract** 
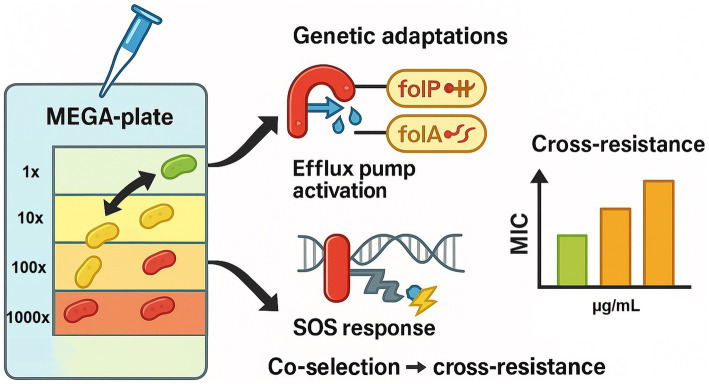

## Introduction

The emergence of antibiotic-resistant bacterial pathogens has rapidly evolved into a global public health concern following the widespread use of antimicrobial therapy. In Gram-negative bacteria in particular, resistance is often attributed to the presence of specific genes associated with mobile genetic elements (1). The spread of multidrug-resistant (MDR) Gram-negative strains is now regarded as one of the most pressing global health threats (2).

Since their introduction in 1935, sulfonamides have been widely used for the treatment of infections caused by both Gram-positive and Gram-negative bacteria, as well as protozoa. Due to the rapid emergence of resistance, sulfonamides have typically been administered in combination with diaminopyrimidines — most commonly trimethoprim — since the 1970s (3, 4). In veterinary medicine, they are primarily used for the prevention and treatment of enteric diseases, either alone or in combination with other antimicrobials (5).

Sulfonamides, structural analogs of para-aminobenzoic acid (PABA), inhibit dihydropteroate synthase (DHPS), a key folate biosynthesis enzyme (6). In *Escherichia coli*, resistance to sulfonamides can arise through chromosomal mutations in the *folP* gene (7, 8), but more commonly results from the acquisition of alternative *sul* genes encoding variant DHPS enzymes with reduced affinity for sulfonamides (9, 10). In the second step of folate biosynthesis, dihydrofolate reductase (DHFR) catalyzes the reduction of dihydrofolate to tetrahydrofolate using NADPH, and it is at this enzymatic step that diaminopyrimidines exert their inhibitory action (11). DHFR is essential for the biosynthesis of thymidylate, purines, and several amino acids, including glycine, methionine, serine, and N-formyl-methionyl-tRNA (12, 13). Inhibition of folate-metabolizing enzymes disrupts thymidylate biosynthesis, impairs DNA replication, and ultimately leads to cell death (14).

Despite their continued importance in clinical settings — particularly for urinary tract infections — and in livestock production, widespread and persistent sulfonamide resistance has been documented globally in Gram-negative strains from both humans and animals (15–17). Pathogenic *E. coli* strains frequently exhibit resistance to sulfonamides (18). Three key genes underlie this resistance: *sul1*, *sul2*, and *sul3* (5, 19). The *sul1* gene is most commonly found on conjugative plasmids and class 1 integrons (17), while *sul2* was initially identified on non-conjugative plasmids but is now increasingly detected on conjugative plasmids, often co-occurring with streptomycin resistance (16). The *sul3* gene was first recorded in bacterial samples taken from pigs in Switzerland in 2003 and has since been identified in both human and animal isolates (20), and is frequently associated with non-classical integrons (5).

Resistance-conferring mutations are often associated with a so-called “fitness cost” syndrome. As many antibiotics target essential cellular functions, the acquisition of resistance may impair bacterial fitness by imposing additional energetic burdens or by interfering with core biological processes (21–23). However, resistance may persist even after the selective pressure has been removed, due to gene linkage, co-selection, or compensatory mutations (24).

*E. coli* was selected as the model organism for this study due to its well-characterized genetics, rapid growth, and wide use in laboratory evolution experiments. As one of the most prevalent Gram-negative facultative anaerobes, *E. coli* frequently serves as a host for the acquisition and dissemination of antimicrobial resistance (AMR) genes, including plasmid-borne elements. Its ability to readily accumulate and maintain diverse resistance determinants makes it a relevant and representative system for studying adaptive trajectories under antibiotic pressure (23, 25).

Mitigating resistance requires the implementation of preventive strategies, such as adherence to biosecurity protocols (26), and the stabilization of the host microbiome through the use of prebiotics and probiotics (27, 28). Furthermore, antimicrobial stewardship should be supported by the targeted application of antibiotics and, where feasible, by replacing them with alternative agents such as antimicrobial peptides, medium-chain fatty acids, plant extracts, or essential oils (29–35).

The aim of this study was to investigate the microevolutionary adaptation of *E. coli* to potentiated sulfonamides (trimethoprim-sulfamethoxazole) under *in vitro* conditions, using a controlled model system. Special attention was given to the identification of resistance-related genetic mutations and co-selection mechanisms that may contribute to the emergence of cross-resistance to other antimicrobials.

## Materials and methods

### Bacterial strain

Experiments used the *E. coli* reference strain ATCC 25922, a widely accepted quality control organism for antimicrobial susceptibility testing.

### MEGA-plate preparation

To enable gradient-based selection, a custom MEGA-plate system was designed and constructed from polycarbonate sheets (dimensions: 60 × 30 cm; thickness: 5 mm) by Innoterm Ltd. (Budapest, Hungary). The design was based on the original evolutionary plate format (36), and refined using established protocols (37). For sterilization, a 7.5% hydrogen peroxide solution was prepared by diluting 30% stock hydrogen peroxide (VWR International Ltd., Hungary) with deionized water at a 1:3 ratio, as described in prior studies (38, 39). The plate chamber was placed under a laminar flow hood, filled with the disinfectant, and the inner surface of the lid was wiped with 0.9% sodium hypochlorite (NaOCl) (Merck KGaA, Darmstadt, Germany). After 15 min of exposure, the disinfectant was removed using vacuum suction, and the system was subjected to UV irradiation for 30 min to complete the decontamination process.

The MEGA-plate was assembled in three sequential layers. The growth medium was layered in three distinct phases. The base layer comprised nine separated zones containing incrementally increasing concentrations of the tested antimicrobial compound (0×, 1×, 10×, 100×, and 1,000×), arranged symmetrically from the edges toward the center. This was followed by a continuous solid agar layer that provided structural stability. The uppermost layer consisted of semi-solid agar to allow bacterial movement and gradual migration across the gradient. The antimicrobial concentration increments (0×, 1×, 10×, 100×, and 1,000×) were selected to model large-scale evolutionary transitions and to simulate strong, stepwise selection pressure across several orders of magnitude. This log-scale gradient approach is based on the original MEGA-plate concept introduced by Baym et al. (36), which demonstrated that logarithmic steps (e.g., 10-fold increases) allow observation of successive mutational sweeps and adaptation bottlenecks as bacterial populations advance through increasingly selective environments. Such a design allows the detection of both early and late resistance adaptations, including low-level tolerance mechanisms at 1 × to 10 × MIC, and high-level mutational resistance at extreme concentrations such as 1,000 ×.

Agar was prepared using 2% BD Bacto Agar (VWR International Ltd., Debrecen, Hungary) for all layers, except the top layer which contained 0.28% agar to allow for semi-solid consistency. Each liter of medium was supplemented with one LB-Lennox capsule (VWR International Ltd., Debrecen, Hungary), while the top layer received two capsules to optimize surface growth conditions. Cycloheximide (64 μg/mL final concentration; Merck KGaA, Darmstadt, Germany) was incorporated to inhibit fungal contamination. For enhanced visual contrast during imaging, black acrylic paint (Artmie, Budapest, Hungary) was added at 4 mL/L to the base and intermediate layers.

The *E. coli* inoculum was prepared by adjusting the culture to 0.5 McFarland turbidity (1.5 × 10^8^ CFU/mL) using a nephelometer (Thermo Fisher Scientific, Waltham, MA, United States). The *E. coli* inoculum was applied to both lateral ends of the plate, by dispensing 100 μL of the standardized suspension evenly along the entire edge to form continuous inoculation lines. Inoculation was performed immediately after the top layer had solidified at room temperature. The plate was subsequently incubated at 37 °C.

### Antibiotic susceptibility testing

Antimicrobial susceptibility was initially assessed by determining the MIC of each agent against the *E. coli* ATCC 25922 strain, following the Clinical and Laboratory Standards Institute (CLSI) guidelines (40). To facilitate the evolution of resistant clones, the 1 × baseline concentration of potentiated sulphonamide was defined as one-quarter of the MIC (2 μg/mL), with subsequent concentrations (10×, 100×, and 1,000×) scaled accordingly for MEGA-plate application.

After 10 days of incubation on the MEGA-plate — during which the bacterial population successfully expanded through all concentration gradients including 1,000 × — samples were taken from each antibiotic exposure zone. These were streaked onto ChromoBio Coliform agar (Biolab Zrt., Budapest, Hungary), a chromogenic selective medium, to confirm the absence of cross-contamination. Single colonies were subcultured onto tryptic soy agar and cryopreserved in Microbank vials at −80 °C for later analysis.

Three independent biological replicates were performed for each antibiotic concentration, using separate MEGA-plate assays. For each replicate, samples were collected from three parallel locations within the gradient region of interest. These parallel samples were not treated as technical replicates, as they originated from distinct spatial points and served to confirm intra-experimental consistency. All recovered isolates from a given MEGA-plate exhibited uniform susceptibility profiles. Frozen stocks were revived 24 h prior to testing by inoculating into 3 mL of cation-adjusted Mueller-Hinton broth (CAMHB; Biolab Zrt., Budapest, Hungary), followed by incubation at 37 °C for 18–24 h.

Broth microdilution assays were conducted in 96-well microtiter plates. Wells in columns 2 through 12 were filled with 90 μL of CAMHB (Step 1). Stock solutions of the test compounds — ceftriaxone, cefquinome, cefotaxime, ceftiofur, colistin, enrofloxacin, amoxicillin, neomycin, oxytetracycline, florfenicol, and potentiated sulfonamide (trimethoprim-sulfamethoxazole, 1:19) — were prepared at 1024 μg/mL according to CLSI standards, diluted 1:1 in CAMHB, and 180 μL of each solution was dispensed into column 1 (Step 2). Twofold serial dilutions were performed across the plate (Step 3). To standardize volume, pipette tips were discarded after column 10, ensuring a consistent 90 μL final volume in each well.

Bacterial suspensions were adjusted to a 0.5 McFarland standard using a nephelometer (ThermoFisher Scientific, Budapest, Hungary), and 10 μL of each suspension was added to the wells in reverse order from column 11 to column 2 (41). MIC endpoints were determined and analyzed using standardized methods.

### Extended-spectrum beta-lactamase (ESBL) detection

Extended-spectrum beta-lactamase (ESBL) activity in *E. coli* isolates was assessed per CLSI protocols (40). MICs were determined for ceftazidime and cefotaxime, both alone and in combination with clavulanic acid, a *β*-lactamase inhibitor. A fixed clavulanic acid concentration of 4 μg/mL was maintained across all wells containing the antibiotic-inhibitor combinations.

To prepare the inhibitor solution, 10 mg of clavulanic acid was dissolved in 10 mL of distilled water and filtered through a sterile cellulose membrane. This solution was then mixed with 230 mL of sterile cation-adjusted Mueller-Hinton broth (CAMHB; Merck KGaA, Darmstadt, Germany), producing a final concentration equivalent to 10 mg per 240 mL.

For the working plates, wells designated for ESBL testing were pre-filled with 240 μL of this clavulanate-supplemented broth. A 10 μL aliquot of bacterial suspension was added to each well, followed by a 10 μL transfer into 90 μL of CAMHB in the test plates, resulting in a precise final clavulanic acid concentration of 4 μg/mL per well.

Plates were incubated at 37 °C for 18–24 h. Interpretation of results followed CLSI standards: an isolate was considered ESBL-positive if the MIC of the beta-lactam agent decreased by threefold or more in the presence of clavulanic acid. For example, a reduction in ceftazidime MIC from 8 μg/mL to 1 μg/mL in the clavulanic acid combination would indicate ESBL production.

### Molecular genetic analysis

To assess the genomic effects of antibiotic exposure and screen for genes potentially involved in ESBL production, we performed comprehensive molecular genetic analyses. Genomic DNA was extracted using the Zymo Quick-DNA Fungal/Bacterial Miniprep Kit, per manufacturer’s instructions. Cell lysis and mechanical disruption were achieved by bead beating using a Qiagen TissueLyzer LT system (Qiagen GmbH, Hilden, Germany) at 50 Hz for 5 min. Extracted nucleic acids were stored at −20 °C until further use.

Sequencing was conducted on an Illumina NextSeq 500 platform, employing paired-end reads. Illumina’s sequencing-by-synthesis technology involves immobilization of DNA fragments via bridge amplification, removal of the reverse strand, and iterative nucleotide incorporation with fluorescent signal detection (42, 43).

For co-evolutionary assessments and comparative genomic analysis, whole-genome libraries were prepared using the Illumina^®^ Nextera XT DNA Library Preparation Kit (Illumina, San Diego, USA). Fragment-specific dual-index barcoding was achieved using Nextera XT Index Kit v2 Set A. DNA input was standardized to 0.2 ng/μL in a final volume of 2.5 μL. This was mixed with 5 μL of Tagment DNA buffer and 2.5 μL of Amplicon Tagment Mix and incubated at 55 °C for 6 min using an Eppendorf Mastercycler nexus GX2 thermal cycler (Eppendorf SE, Hamburg, Germany). The reaction was cooled to 10 °C and neutralized with 2.5 μL of Neutralize Tagment buffer, followed by a 5-min room-temperature incubation.

For library amplification, 7.5 μL of Nextera PCR Master Mix was combined with 2.5 μL each of i5 and i7 index primers and added to the tagmented DNA. PCR conditions included an initial denaturation at 95 °C for 30 s, followed by 12 cycles (95 °C for 10 s, 55 °C for 30 s, 72 °C for 30 s), and a final extension at 72 °C for 5 min. Reactions were cooled to 10 °C. Libraries were purified using the Gel/PCR DNA Fragments Extraction Kit (Geneaid Biotech, Hsinchu, Taiwan) according to the column-based protocol, and DNA concentrations were quantified using the Qubit® dsDNA HS Assay Kit (Thermo Fisher Scientific, Waltham, MA, United States). Indexed libraries were pooled and normalized for sequencing.

To ensure high-confidence variant interpretation, all detected mutations were subjected to quality filtering prior to downstream analysis. Variants were classified as “confidently characterized” if they met the following criteria: (i) a minimum variant allele frequency (VAF) of ≥90%, (ii) a coverage depth of at least 30 × at the mutation site, (iii) consistent detection across at least two of the three biological replicates, and (iv) unambiguous functional annotation by both SnpEff and Prokka pipelines. Variants not fulfilling these thresholds were excluded from quantitative summary statistics and functional interpretation.

## Results

### Antimicrobial resistance gene set

Based on GenomeScope analyses used for general genome characterization, the k-mer frequency histograms consistently aligned with the expected size of *E. coli* genomes (Supplementary Figures 1–5). Furthermore, taxonomic classification performed using CheckM (v1.1.6) and Kraken (v1.1.1.1) confirmed a 100% match with *E. coli* across all samples.

Resistance gene annotation using the Comprehensive Antibiotic Resistance Database (CARD) identified 44 distinct genes with ≥90% sequence identity and coverage [additional data (potentiated sulphonamide)]. The identified ARG profile was identical across all samples and conferred resistance against 22 classes of antibiotics, disinfectants, and dyes; as in our previous similar studies (44, 45). Of particular note, the *ampC* and *ampH* genes — encoding *β*-lactamase enzymes — are critical for resistance to β-lactam antibiotics (e.g., penicillins and cephalosporins) via enzymatic inactivation (Table 1). The presence of *acrA*, *acrB*, and *tolC* genes indicates a functional multidrug efflux pump system, which is commonly found in *E. coli*. Similar efflux systems are formed by the combination of *tolC* with *emrA* and *emrB*, as well as *mdtA*, *mdtB*, and *mdtC* genes.

**Table 1:** The 44 antimicrobial resistance genes identified by next-generation sequencing, consistently detected across all samples.

Gene	C%	I%	Mechanism	Resistance
*acrA*	100.00	99.16	Efflux pump	cephalosporins, fluoroquinolones, glycylcyclines, penicillins, phenicols, rifamycins, tetracyclines, triclosan
*acrB*	100.00	98.64	Efflux pump	
*acrD*	100.00	98.04	Efflux pump	aminoglycosides
*acrE*	100.00	98.79	Efflux pump	cephalosporins, cephamycins, fluoroquinolones, penicillins
*acrF*	100.00	96.49	Efflux pump	
*acrS*	100.00	98.34	Efflux pump	cephalosporins, cephamycins, fluoroquinolones, glycylcyclines, penicillins, phenicols, rifamycins, tetracyclines, triclosan
*ampC*	100.00	98.15	Enzymatic inactivation	cephalosporins, penicillins
*ampH*	100.00	97.50	Enzymatic inactivation	
*bacA*	99.76	98.17	Target mutation	peptide antibiotics
*baeR*	99.86	96.81	Efflux pump	aminocoumarins, aminoglycosides
*baeS*	100.00	90.53	Efflux pump	
*cpxA*	100.00	98.47	Efflux pump	
*CRP*	100.00	99.21	Efflux pump	fluoroquinolones, macrolides, penicillins
*emrA*	100.00	98.21	Efflux pump	fluoroquinolones
*emrB*	100.00	96.95	Efflux pump	
*emrE*	100.00	92.19	efflux pump	macrolides
*emrK*	100.00	97.73	Efflux pump	tetracyclines
*emrR*	100.00	98.68	Efflux pump	fluoroquinolones
*emrY*	100.00	97.73	Efflux pump	tetracyclines
*eptA*	100.00	91.85	Target mutation	peptide antibiotics
*evgA*	100.00	99.02	Efflux pump	fluoroquinolones, macrolides, penicillins, tetracyclines
*evgS*	100.00	96.19	Efflux pump	
*gadW*	100.00	99.86	Efflux pump	fluoroquinolones, macrolides, penicillins
*gadX*	100.00	93.82	Efflux pump	
*H-NS*	100.00	99.28	Efflux pump	cephalosporins, cephamycins, fluoroquinolones, macrolides, penicillins, tetracyclines
*kdpE*	99.26	95.84	Efflux pump	aminoglycosides
*marA*	100.00	98.70	Reduce permeability	carbapenems, cephalosporins, cephamycins, fluoroquinolones, glycylcyclines, monobactams, penicillins, phenicols, rifamycins, tetracyclines, triclosan
*mdfA*	100.00	96.59	Efflux pump	benzalkonium chloride, rhodamine, tetracyclines
*mdtA*	100.00	95.11	Efflux pump	aminocoumarins
*mdtB*	100.00	96.29	Efflux pump	
*mdtC*	100.00	94.15	Efflux pump	
*mdtE*	100.00	98.62	Efflux pump	fluoroquinolones, macrolides, penicillins
*mdtF*	100.00	97.33	Efflux pump	
*mdtG*	100.00	98.21	Efflux pump	fosfomycin
*mdtH*	100.00	98.26	Efflux pump	fluoroquinolones
*mdtM*	100.00	95.05	Efflux pump	acridines, fluoroquinolones, lincosamides, nucleosides, phenicols
*mdtN*	100.00	95.64	Efflux pump	acridines, nucleosides
*mdtO*	100.00	97.08	Efflux pump	
*mdtP*	100.00	97.61	Efflux pump	
*msbA*	100.00	98.06	Efflux pump	nitroimidazoles
*pmrF*	100.00	97.63	Target mutation	peptide antibiotics
*tolC*	100.00	97.98	Efflux pump	aminocoumarins, aminoglycosides, carbapenems, cephalosporins, cephamycins, fluoroquinolones, glycylcyclines, macrolides, penicillins, peptide antibiotics, phenicols, rifamycins, tetracyclines, triclosan
*ugd*	100.00	96.92	Target mutation	peptide antibiotics
*yojI*	100.00	98.05	Efflux pump	

C – coverage; I – identity.

**Figure 1:**
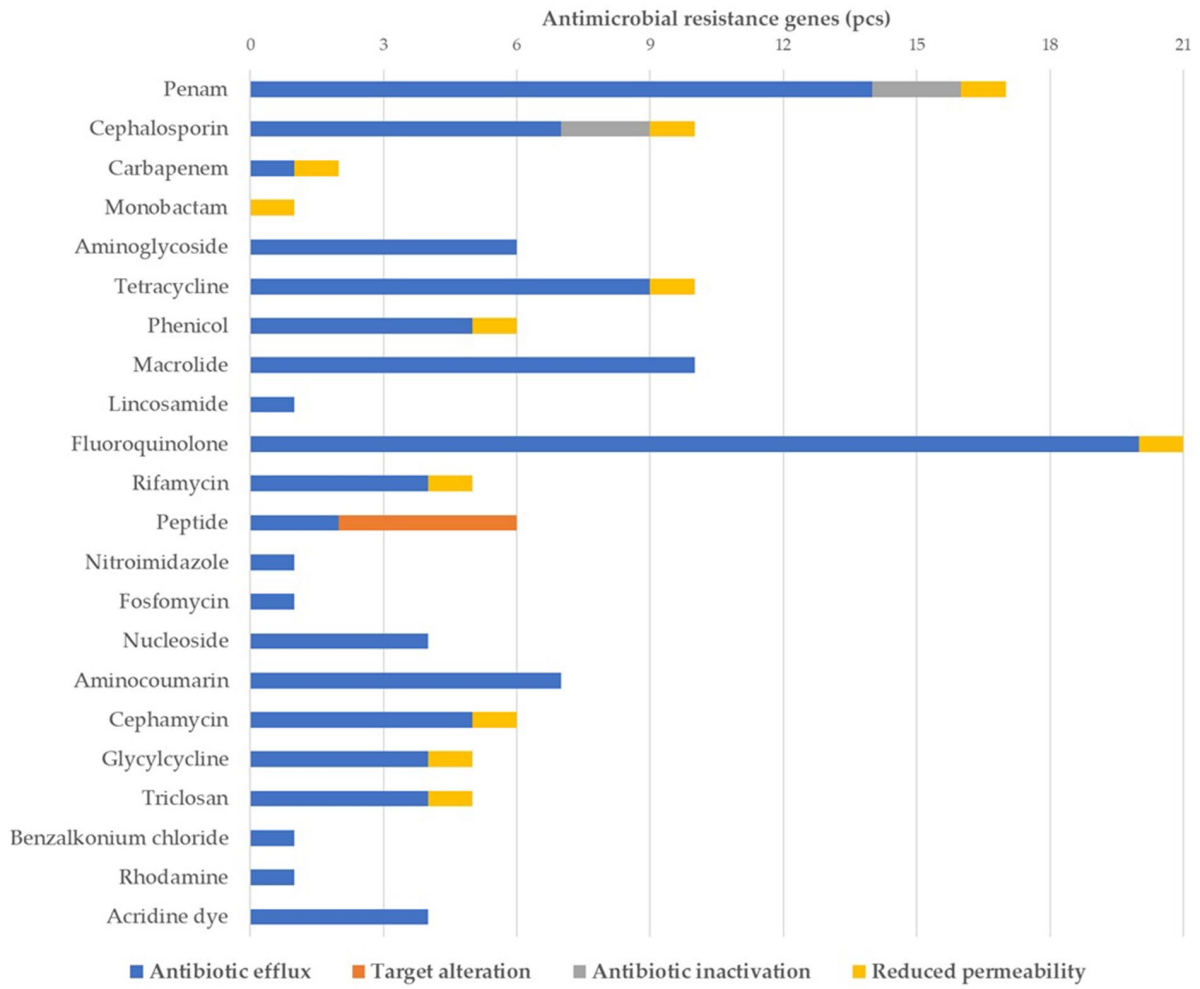
Classification of the identified antimicrobial resistance genes by antimicrobial class and resistance mechanism.

Figure 1 presents the classification of the 44 identified ARGs according to drug class and resistance mechanism. Of these, 21 genes confer fluoroquinolone resistance, with 19 encoding efflux pumps. The second most prevalent group includes genes conferring resistance to penicillins (*n* = 17), of which 14 are efflux pump-related and two (*ampC*, *ampH*) are associated with enzymatic inactivation. Target modification resistance genes (*n* = 4) were exclusively associated with peptide (polymyxin) antibiotics.

**Figure 2:**
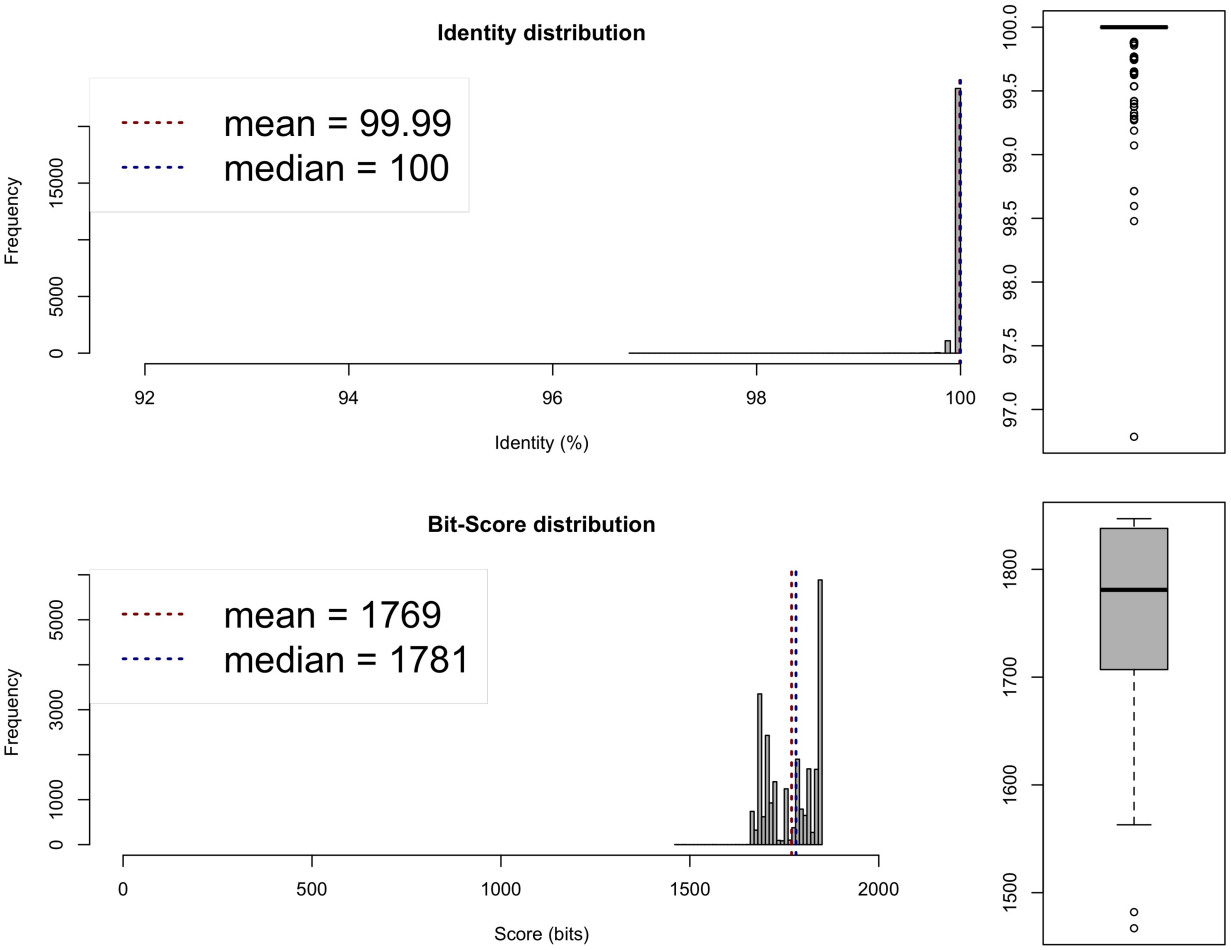
Average nucleotide identity (ANI) analysis between the genomes of the 0 × and 1,000 × potentiated sulphonamide-exposed samples, showing species-level similarity exceeding 95%. One-way ANI 1: 99.98% (SD: 0.44%), based on 24,651 fragments. One-way ANI 2: 99.99% (SD: 0.11%), based on 24,584 fragments. Two-way ANI: 99.99% (SD: 0.04%), based on 24,544 fragments.

The *folP* gene, associated with sulfonamide resistance, was detected but did not meet the CARD inclusion threshold. However, the identification of point mutations in *folP* suggests a potential key role in the observed resistance phenotype to potentiated sulfonamides (see Figure 2).

Based on the sequencing data, serotyping of the studied strain was also possible, leading to the identification of O6 type-specific polysaccharide antigens (*wzx, wzy*) and H1 and H12 protein antigens (*fliC*).

### Phenotypic results

When exposed to potentiated sulphonamide, bacteria grew across the highest antibiotic concentration within 7 days. Exposure to potentiated sulphonamide resulted in increased MIC values for all tested antibiotics at both 100 × and 1,000 × concentrations (Table 2).

**Table 2:** Effect of increasing potentiated sulphonamide concentrations on the minimum inhibitory concentration (MIC) values of the tested antimicrobial agents.

Sample	PSA	CTX	CFR	CFT	CFQ	ENR	COL	OTC	AMX	FLO	NEO
μg/mL											
0 × PSA	8	0.03	0.25	0.06	0.06	0.003	0.5	2	8	16	16
1 × PSA	8	0.03	**0.5**	0.06	0.06	0.003	0.5	2	8	16	**32**
10 × PSA	**256**	**1**	**1**	**0.25**	**0.25**	**0.06**	0.5	2	**32**	**128**	**32**
100 × PSA	**512**	**2**	**1**	**0.5**	**0.5**	**0.06**	**32**	**4**	**64**	**256**	**64**
1,000 × PSA	**>512**	**2**	**2**	**4**	**1**	**0.5**	**32**	**16**	**64**	**512**	**64**

Bolded values indicate elevated MICs (μg/mL) compared to the baseline. Red boxes highlight critically important antimicrobials for public health (AMEG category B). PSA – potentiated sulphonamide (trimethoprim and sulfamethoxazole in a 1:19 ratio), CTX – cefotaxime, CFR – ceftriaxone, CFT – ceftiofur, CFQ – cefquinome, ENR – enrofloxacin, COL – colistin, OTC – oxytetracycline, AMX – amoxicillin, FLO – florfenicol, NEO – neomycin.

Across all three biological replicates, MIC values remained consistent, with no detectable variation between corresponding concentration endpoints. Intra-plate sampling from three parallel locations yielded identical susceptibility profiles, indicating high spatial uniformity. Given the full agreement among replicates, basic descriptive statistics (mean ± SD) are not presented for MIC changes, as standard deviation was effectively zero across all measurements. These results confirm the robustness and reproducibility of resistance acquisition patterns under sulphonamide exposure.

ESBL production assays yielded negative results (see Table 3).

**Table 3:** Results of the extended-spectrum β-lactamase (ESBL) test using ceftazidime (CTZ), cefotaxime (CTX), and their respective clavulanic acid (KLA) combinations, following the guidelines of the Clinical Laboratory Standards Institute (CLSI).

Sample	CTZ	CTZ + CLA	Difference	CTX	CTX + CLA	Difference
	(μg/mL)			(μg/mL)		
0 × PSA	0.03	0.03	1×	0.03	0.03	1×
1 × PSA	0.03	0.03	1×	0.03	0.03	1×
10 × PSA	0.5	0.25	2×	1	0.5	2×
100 × PSA	0.5	0.25	2×	2	2	1×
1,000 × PSA	0.5	0.5	1×	2	2	1×

PSA — potentiated sulphonamide (trimethoprim and sulfamethoxazole in a 1:19 ratio), AMX – amoxicillin, CTZ – ceftazidime, CTZ + KLA – ceftazidime with clavulanic acid, CTX – cefotaxime, CTX + KLA – cefotaxime with clavulanic acid.

### Genotypic results

All contigs passed quality control, ensuring consistent sequencing quality (see Table 4).

**Table 4:** Contig quality metrics based on QUAST software analysis for potentiated sulphonamide-exposed samples.

Sample	Read	Contigs	Coverage	N_50_	N_75_	L_50_	L_75_
0 × PSA	3,362,592	115	164.453	192,736	107,365	7	16
1 × PSA	3,600,632	124	169.511	202,436	107,365	8	17
10 × PSA	2,992,404	130	166.197	184,845	111,826	10	18
100 × PSA	3,978,966	144	160.161	144,711	813,68	12	23
1,000 × PSA	3,099,440	131	145.395	160,547	104,931	10	20

PSA — potentiated sulphonamide (trimethoprim and sulfamethoxazole in a 1:19 ratio). N50/N75 – the length of the shortest contig such that 50%/75% of the total genome length is contained in contigs of this length or longer; L50/L75 – the minimum number of contigs whose cumulative length accounts for 50%/75% of the total genome assembly length.

The *bacA* gene was detected on phage elements in all samples. The *emrB* gene was found on phage-associated sequences in both 10 and 1,000 potentiated sulphonamide-treated samples. Additionally, *bacA* was identified as a mobile genetic element (MGE) in all cases, while *acrE* and *acrF* were present as MGEs in the 1 × and 10 × samples.

The number of mutations detected per sample compared to the reference genome is summarized in Table 5. A total of 9,278 mutations were identified; 4,935 were classified with confidence (criteria detailed in Methods). The distribution of total mutations ranged from 1,752 to 1,958 per sample, while the number of identified mutations ranged from 923 to 1,074. Mutation overlap with the 0 baseline strain was: 1 sample, 101.6; 10 sample, 107.3; 100 sample, 109.4; and 1,000 sample, 116.4.

**Table 5:** Number of total and identified mutations per sample, classified by mutation type.

Type of mutation		0 × PSA	1 × PSA	10 × PSA	100 × PSA	1,000 × PSA
Complex*	Identified	118	122	115	115	117 (+1)
	All	293	310	300	286	294
Deletion	Identified	20	20	21 (+3)	27 (+3)	29 (+11)
	All	42	42	48	56	68
Inversion	Identified	4	4	12 (+8)	14 (+10)	15 (+12)
	All	15	14	23	27	25
SNP**	Identified	781	792 (+1)	842 (+54)	854 (+82)	913 (+114)
	All	1,402	1,465	1,510	1,487	1,571

The values in parentheses show the number of point mutations identified compared to the 0 × PSA sample. *Complex mutation involving multiple insertions, deletions, and substitutions. **Single nucleotide polymorphism (SNP). ENR – enrofloxacin. PSA — potentiated sulphonamide (trimethoprim and sulfamethoxazole in a 1:19 ratio).

Table 6 summarizes the mutations that may have contributed to the phenotypic expression of antimicrobial resistance. An *emrR* gene deletion occurred only in the 1,000 concentration sample. This mutation may promote the expression of the *emrAB-tolC* multidrug efflux pump system, which is known to play a key role in resistance development (46). The *mdtM* gene encodes a multidrug resistance protein shown to interact with the *acrAB-tolC* efflux system. A deletion in *mdtM* was also identified in the 1,000 × treated sample. This gene encodes a transmembrane drug/H^+^ antiporter, which has been implicated in resistance to multiple antibiotics in *E. coli* (47). Additionally, *yfiB*, encoding the *ompA* outer membrane lipoprotein (48), also harbored a deletion in the 1,000 × sample. Mutations in *yfiB* have previously been associated with resistance phenotypes in *E. coli* (49, 50).

**Table 6:** Deletions, inversions, and single nucleotide polymorphisms (SNPs) detected in genes relevant to antimicrobial resistance.

Gene	0x	1x	10x	100x	1,000x	Nucleic acid	Effect	Product
Deletion								
*emrR*					x	GA-G	Frameshift c.291delA p.Lys97fs	Repressor of *emrAB* multidrug efflux pump
*mdtM*					x	CA-C	Frameshift c.15delT p.Phe5fs	MFS-type multidrug efflux transporter
*yfiB*					x	GA-G	Frameshift c.405delA p.Lys135fs	*ompA-family outer membrane protein*
Inversion								
*acrF*					x	T-TC	Frameshift c.1281dupC p.Arg428fs	RND-type efflux permease subunit (*acrEF*-*tolC*)
*mdtF*				x		G-GT	Frameshift c.33dupT p.Ala12fs	RND-type multidrug efflux permease
SNP*								
*acrB*		x	x	x	x	T-C	Frameshift c.985A > G p.Thr329Ala	RND-type efflux pump (*acrAB*-*tolC*) permease
						A-G	Frameshift c.1186 T > C p.Phe396Leu	
						A-G	Frameshift c.95 T > C p.Val32Ala	
						T-C	Frameshift c.1703A > G p.Asp568Gly	
*acrR*				x		A-T	Frameshift c.557A > T p.Asp186Val	Repressor of *acrAB*-*tolC* multidrug pump
*folA*					x	C-T	Frameshift c.62C > T p.Pro21Leu	Type III dihydrofolate reductase
*folP*			x	x	x	C-T	Frameshift c.674G > A p.Gly225Glu	Dihydropteroate synthase (sulfonamide target)
						G-A	Frameshift c.190C > T p.Pro64Ser	
						T-C	Frameshift c.184A > G p.Thr62Ala	
*marR*				x		A-G	Frameshift c.254A > G p.Glu85Gly	Multidrug resistance transcriptional regulator
*mdtB*				x		T-C	Similar variant c.2883 T > C p.Cys961Cys	RND-type multidrug efflux pump (permease)
*mdtF*					x	A-G	Frameshift c.2510A > G p.Asp837Gly	RND-type multidrug efflux pump component

*Single nucleotide polymorphism.

An inversion in the *acrF* gene was observed in the 1,000 × sample. *acrF* encodes an inner membrane transporter functionally analogous to *acrB*, and is a component of the *acrEF-tolC* efflux pump complex, which plays a key role in multidrug efflux (51). The *mdtF* gene showed both a deletion and a single nucleotide polymorphism (SNP) in the 100 × sample. This gene encodes the inner membrane transporter of the *mdtEF-tolC* multidrug efflux system (52).

Among the identified SNPs, *acrB* mutations were detected at 1, 10, 100, and 1,000 concentrations; these merit particular attention. *acrB* is a key subunit of the *acrAB-tolC* efflux complex, functioning as a heterotrimeric drug-proton antiporter across the inner membrane (53–56). Notably, *acrR*—the *acrAB-tolC* repressor—mutated in several samples. These changes are known to result in high-level antibiotic resistance (57).

For potentiated sulphonamides, *folA* mutation was exclusive to the 1,000 sample, while *folP* mutations occurred at 10, 100, and 1,000. The *folA* encodes dihydrofolate reductase, a key enzyme in the synthesis of tetrahydrofolate, which is essential for the *de novo* synthesis of the DNA nucleotide thymidine. One known bacterial resistance mechanism is the overproduction of this enzyme, and the observed *folA* mutation may reflect such adaptation to trimethoprim exposure (58).

In the 100 sample, a *marR* mutation may have upregulated *marA*, activating *acrAB-tolC* efflux (59, 60).

An SNP in *mdtB* (*mdtABC-tolC* system) was found in the 100 sample. Loss-of-function mutations in *mdtB* can lead to compensatory expression of *mdtC*, preserving pump activity (61).

An additional SNP in *mdtF* was identified in the 1,000 × sample, potentially enhancing activity of the *mdtEF-tolC* system (52), which may explain the observed MIC increases for multiple antibiotics.

Potentiated sulphonamide exposure induced diverse mutations, especially in efflux regulation and folate metabolism genes. These genetic alterations likely underpin the observed increases in MIC not only to sulfamethoxazole and trimethoprim, but to other unrelated antibiotics as well. Furthermore, mutations were detected in key SOS-box regulatory genes—including *recN*, *recQ*, and *uvrB*—in the 100 × and 1,000 × samples, suggesting an induced stress response pathway.

## Discussion

Bacterial resistance to sulfonamides usually involves gene mutation or gene substitution. In the mutation-based mechanism, alterations in the chromosomal *folP* gene, which encodes DHPS, confer resistance to sulfonamides (24). In the substitution-based pathway, sulfonamide resistance arises from the acquisition of alternative DHPS-encoding genes (*sul1*, *sul2*, *sul3*), which produce enzymes with lower affinity for sulfonamides. This latter mechanism is more prevalent in clinical and environmental isolates (62, 63).

Sulfonamides, competitive inhibitors of DHPS, block PABA conversion to dihydropteroate, disrupting folate biosynthesis. Folates function as essential cofactors in nucleotide and certain amino acid biosynthesis. Consequently, DHPS inhibition blocks bacterial proliferation, particularly in organisms unable to scavenge folates from their environment. However, *sul* genes encode DHPS variants that are not inhibited by sulfonamides, thereby conferring resistance (64–67).

Among the genetic changes induced by potentiated sulphonamide exposure, the most critical were mutations observed in the *folP* gene (in 10×, 100×, and 1,000 × samples) and in the *folA* gene (in the 1,000 × sample). The *folA* gene regulates the synthesis of DHFR, which converts dihydrofolate into tetrahydrofolate — a coenzyme essential for *de novo* thymidine nucleotide biosynthesis. One of the known bacterial resistance mechanisms involves overexpression of this enzyme, and the observed *folA* mutation may reflect an adaptation to reduce susceptibility to the diaminopyrimidine component of the drug combination (58).

Meanwhile, *folP* encodes DHPS itself, and point mutations in this gene can impair sulfonamide binding, thereby maintaining folate synthesis and promoting resistance (7). The combined mutations in *folA* and *folP* likely explain the elevated MIC values observed in response to potentiated sulphonamide exposure.

Multidrug efflux transporters are widespread and play a central role in antibiotic resistance mechanisms, as they enable bacteria to evade the action of many current therapeutic agents (68, 69). Among them, ATP-binding cassette (ABC) transporters are essential molecular transport systems that rely on ATP hydrolysis to provide the energy required for translocating various biomolecules across cellular membranes (69). Alterations in ABC transporter function are closely associated with the physiological state of the bacterial strain and environmental growth conditions (24).

Our results suggest that the increased MIC values observed for several antibiotics may be attributed to the activation of efflux pump systems. A deletion in the *emrR* gene, observed exclusively in the 1,000 × PSA-treated strain, could lead to upregulation of the *emrAB-tolC* multidrug efflux pump (46). The *mdtM* gene, which regulates the *acrAB-tolC* efflux system, also showed a deletion in the 1,000 × sample. This gene encodes a transmembrane drug/H^+^ antiporter involved in resistance against multiple agents (47). An inversion in the *acrF* gene, a homolog of *acrB* that contributes to the *acrEF-tolC* efflux complex, was also detected in the 1,000 × strain (51).

Additionally, a mutation in the *acrR* gene — encoding a repressor of the *acrAB-tolC* system—was associated with high-level resistance phenotypes (57). The *marR* gene, whose mutation was observed in the 100 × sample, triggers the expression of the *marA* activator protein, leading to enhanced *acrAB-tolC* pump activity (59, 60).

We also observed a deletion and SNP in the *mdtF* gene (100 × and 1,000 × samples), which encodes the inner membrane transporter of the *mdtEF-tolC* efflux system (52). Moreover, an SNP in *mdtB*, a component of the *mdtABC-tolC* pump, was found in the 100 × sample. If this mutation disrupts *mdtB* expression, it may indirectly activate *mdtC* expression, thus maintaining efflux pump functionality (61).

Several identified mutations occurred in well-characterized efflux regulators, including *emrR*, *marR*, and *acrR*. These genes encode local or global transcriptional repressors that modulate multidrug resistance efflux systems such as EmrAB, AcrAB-TolC, and others. Loss-of-function mutations in *marR* or *acrR* are known to derepress expression of their corresponding operons (*marA* and *acrAB*, respectively), leading to upregulation of the AcrAB-TolC efflux pump and increased tolerance to structurally unrelated antibiotics, including fluoroquinolones, chloramphenicol, and tetracyclines (60, 70). Similarly, inactivation of *emrR* results in overexpression of the EmrAB pump, which has been implicated in resistance to nalidixic acid and other hydrophobic agents (46). These regulatory disruptions likely contribute to the observed cross-resistance phenotypes in our evolved populations, highlighting the role of efflux derepression as a collateral pathway of adaptation under sulphonamide pressure.

These regulatory disruptions likely contribute to the observed cross-resistance phenotypes in our evolved populations, highlighting the role of efflux depression as a collateral pathway of adaptation under sulphonamide pressure. Comparable cross-resistance patterns have been reported in systems exposed to aminoglycosides and fluoroquinolones, where *marR* and *acrR* mutations similarly led to broad-spectrum efflux activation and multidrug tolerance (71, 72).

As for SOS-box related genes, point mutations were identified in *recN*, *recQ*, and *uvrB* in both 100 × and 1,000 × PSA-treated samples. These genes are involved in DNA damage response and may contribute to mutagenesis and adaptive stress responses under antimicrobial pressure.

Horizontal gene transfer (HGT) can occur under a variety of laboratory conditions and can be facilitated by physical means that enhance DNA exchange — such as immobilization on membranes or agar surfaces — and likely by various environmental factors that promote gene acquisition. In our study, *E. coli* strains exposed to increasing concentrations of PSA under controlled conditions accumulated point mutations, confirming the utility of the MEGA-plate system in capturing microevolutionary dynamics (36, 37).

Similar approaches have been successfully used to monitor evolutionary trajectories in response to diverse antimicrobials, including trimethoprim (36), and ciprofloxacin (73), consistently demonstrating that spatial drug gradients can unveil stepwise resistance acquisition and collateral adaptation patterns.

Compared to our previous investigations using *β*-lactams and florfenicol (44, 45), the present study demonstrates that sulphonamides induce a markedly different adaptive landscape. Unlike β-lactam exposure, which primarily selects resistance via target modification or efflux, sulphonamide exposure potentiates hypermutation. This is likely mediated through indirect interference with nucleotide synthesis, leading to replication stress and increased SOS response activation. This mechanistic divergence is critical, while *β*-lactams often select for resistance mutations within the target genes or membrane-associated functions, sulphonamides appear to globally elevate the mutation rate, possibly accelerating the emergence of *de novo* resistance mutations across unrelated loci. Such behavior highlights a previously underappreciated evolutionary pathway, in which sub-inhibitory sulphonamide concentrations function not only as selective agents, but also as mutagenic potentiators, thereby expanding the evolutionary trajectories available to bacterial populations under antimicrobial pressure.

Similar mutagenic effects have been documented for fluoroquinolones and β-lactams under subinhibitory conditions, where they activate the SOS response and drive error-prone polymerase expression (74, 75), albeit through distinct primary mechanisms. Our findings suggest that sulphonamides may exploit a comparable yet broader pathway via nucleotide pool disruption.

It is worth emphasizing that antibiotics — even at subinhibitory concentrations — can promote the emergence of resistance (76). They have been shown to enhance gene transfer and recombination events (77), partially through the activation of the bacterial SOS response (78, 79). These factors may significantly contribute to elevated gene exchange rates in environments such as farms, hospitals, and sewage systems, which offer ideal conditions for the acquisition and dissemination of resistance genes.

## Conclusion

The results of this study confirm that potentiated sulfonamides—specifically the combination of sulfamethoxazole and trimethoprim—can induce substantial genetic adaptation in *E. coli* strains after short-term *in vitro* exposure. Mutations in *folP* and *folA* likely cause resistance to target compounds; efflux pump gene alterations contributed to broad-spectrum cross-resistance. Several of these genetic changes were associated with mobile genetic elements, indicating a potential for horizontal gene transfer and the wider dissemination of resistance traits. The MEGA-plate platform proved to be an effective experimental model for tracking co-evolutionary dynamics. Our findings underscore the need for caution in the clinical and veterinary use of antibiotic combinations and highlight the importance of further molecular investigations into the mechanisms underlying co-selection and multidrug resistance.

## Data Availability

The raw data supporting the conclusions of this article will be made available by the authors. The datasets analyzed for this study can be found in the National Library of Medicine: https://www.ncbi.nlm.nih.gov/bioproject/PRJNA1261795, accessed on 12 May 2025.
