# Supplementary material for: Resistance evolution under potentiated sulphonamide pressure in Escherichia coli
Source: Front Vet Sci. 2025 Nov 26;12:1697872. doi: 10.3389/fvets.2025.1697872 (PMC12690934; doi:10.3389/fvets.2025.1697872)
Supplement: Supplementary file 2 [file Data_Sheet_1.docx]

Supplementary Material


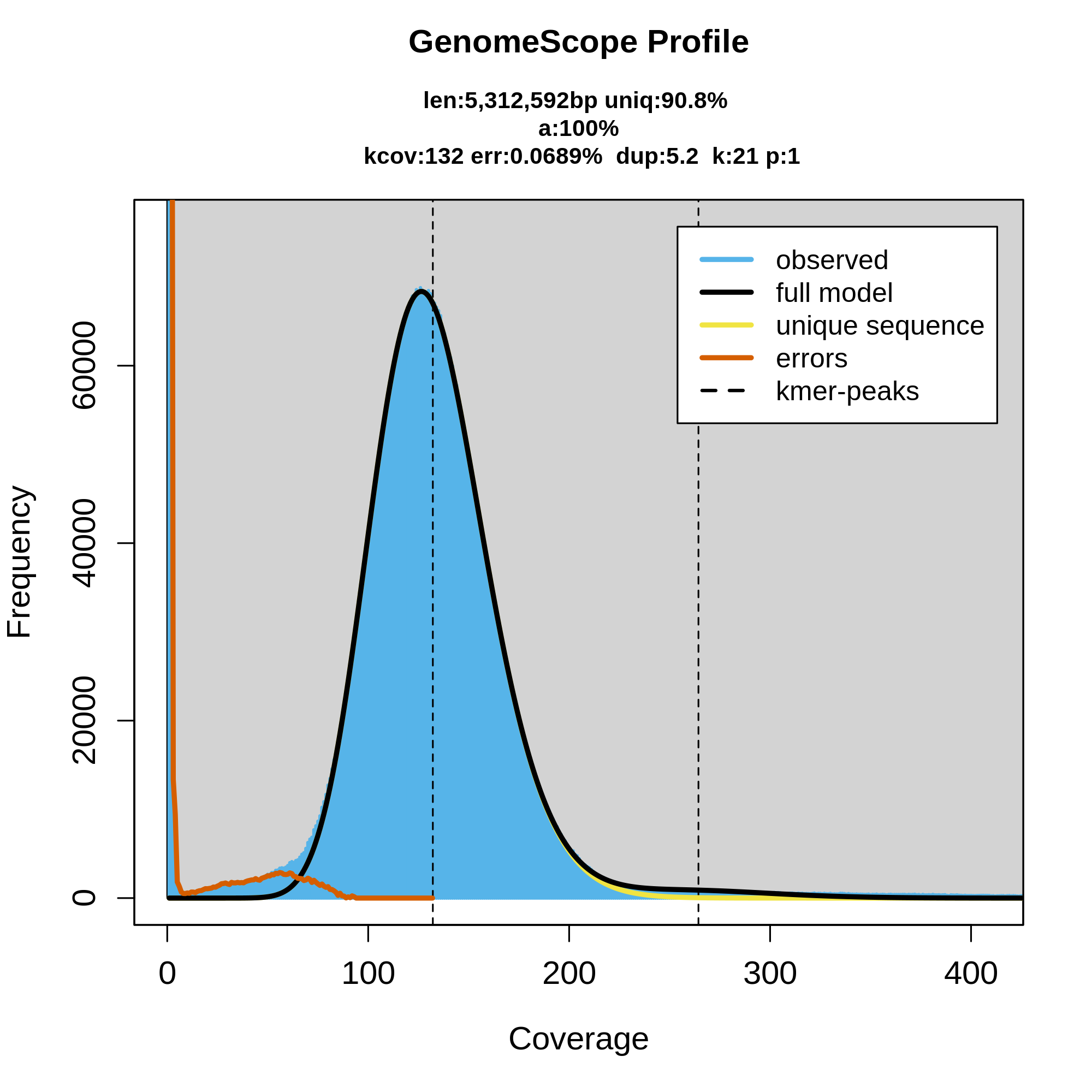
**Supplementary Figure 1** GenoScope profile of 0× PSA sample. The fit of the model (black line) to the observed kmer fre-quencies (blue area) is shown for *Escherichia coli* sample. The genome size, heterozygote ratio and repetitive content of unprocessed short reads can be read.


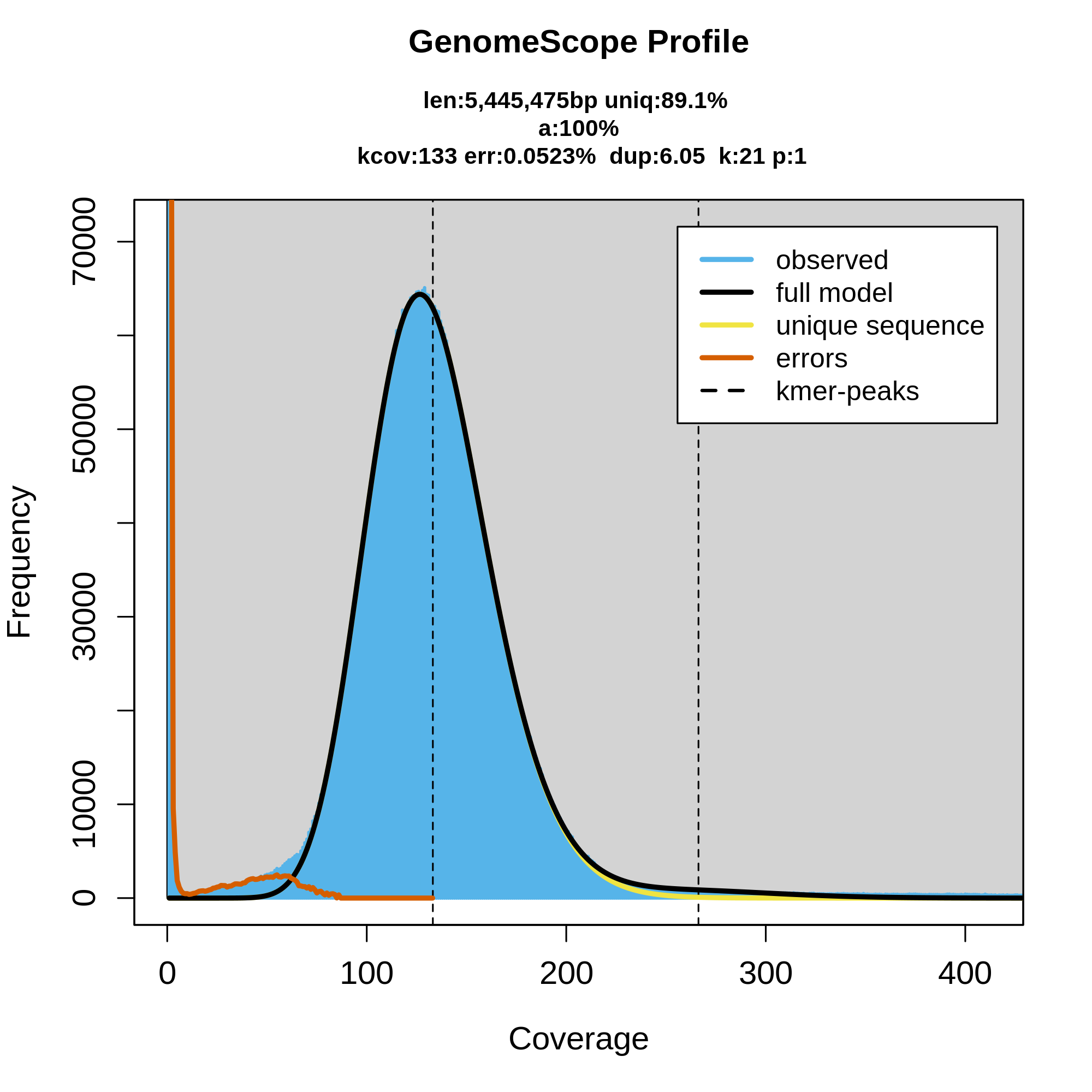
**Supplementary Figure 2** GenoScope profile of 1× PSA sample. The fit of the model (black line) to the observed kmer fre-quencies (blue area) is shown for *Escherichia coli* sample. The genome size, heterozygote ratio and repetitive content of unprocessed short reads can be read.


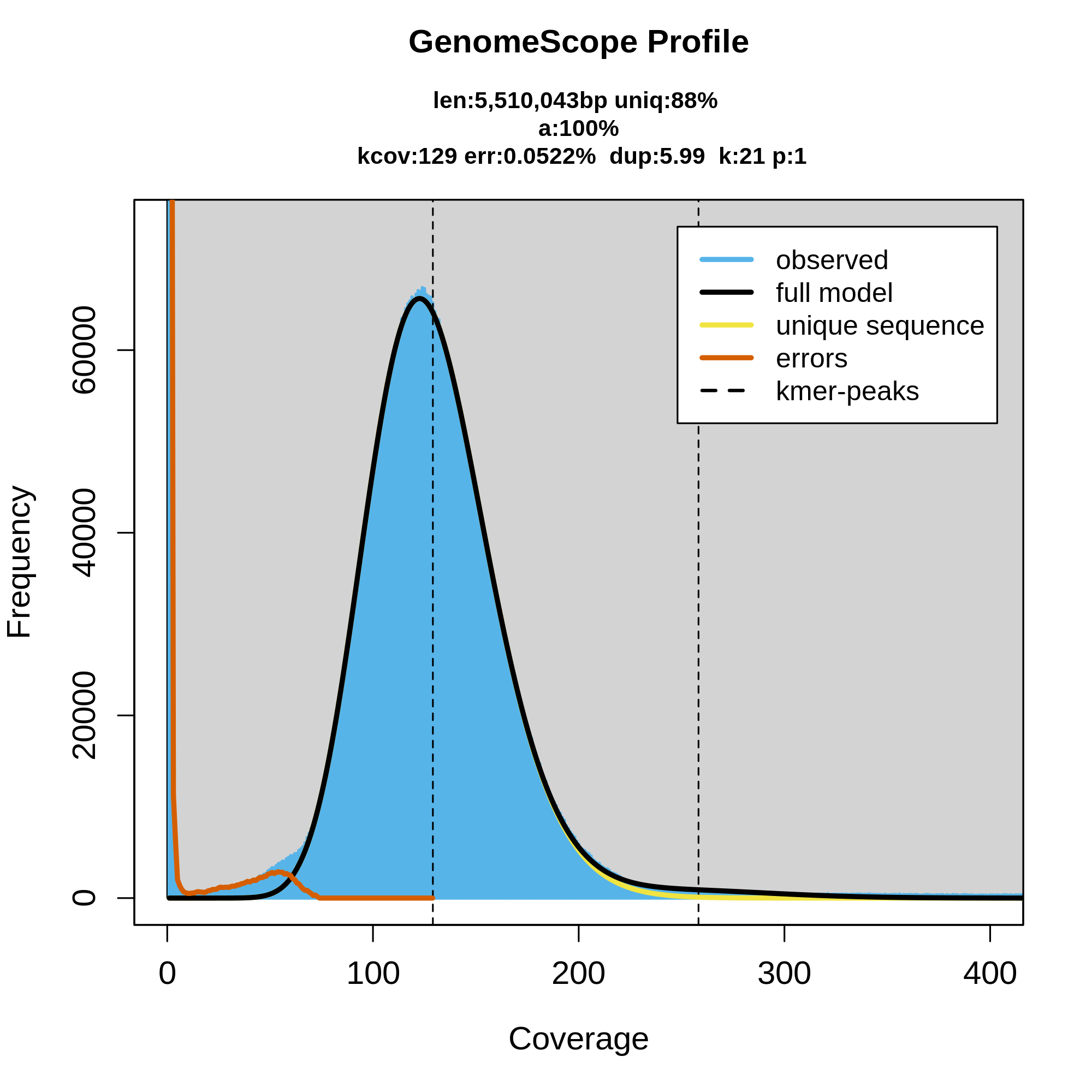
**Supplementary Figure 3** GenoScope profile of 10× PSA sample. The fit of the model (black line) to the observed kmer fre-quencies (blue area) is shown for *Escherichia coli* sample. The genome size, heterozygote ratio and repetitive content of unprocessed short reads can be read.


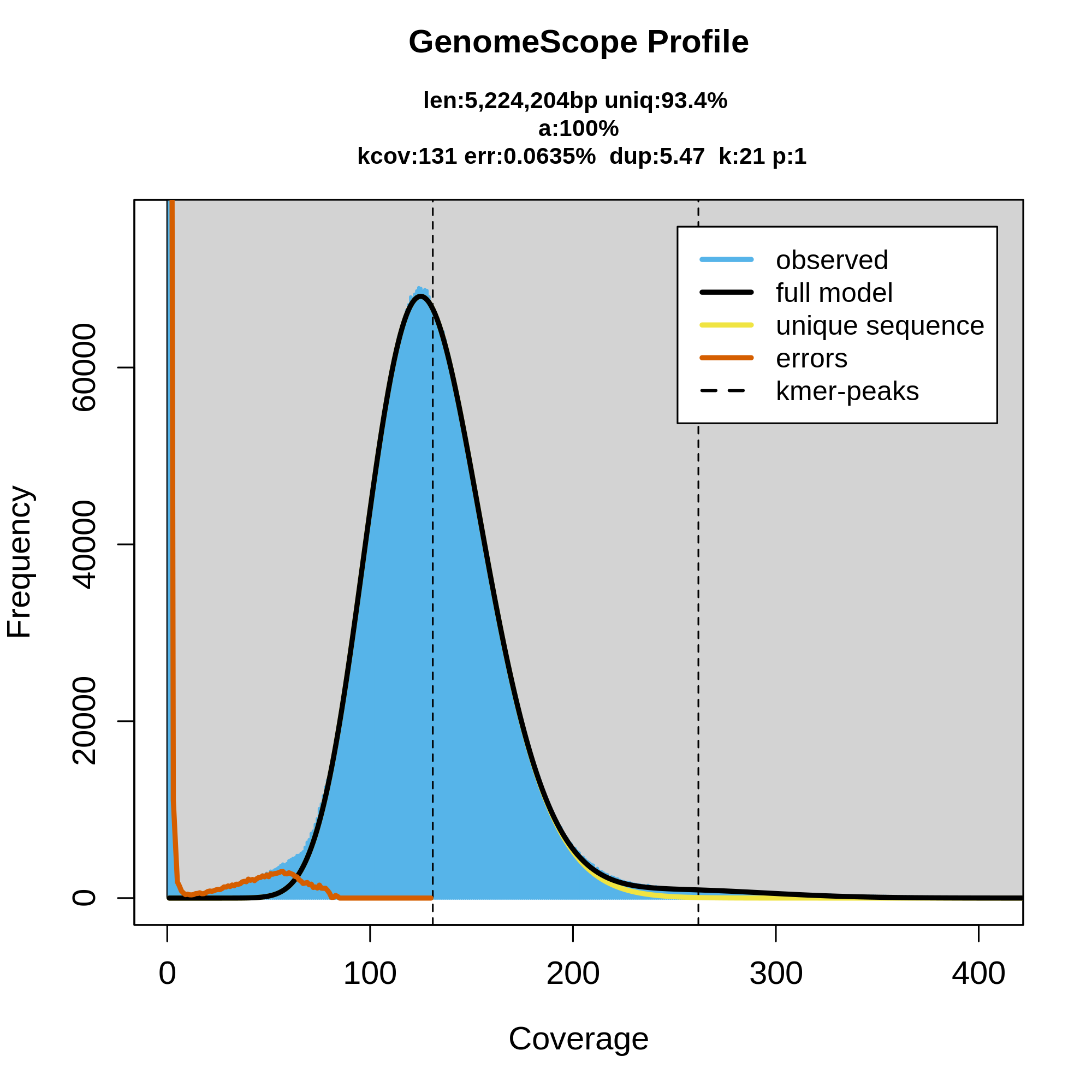


**Supplementary Figure 4** GenoScope profile of 100× PSA sample. The fit of the model (black line) to the observed kmer fre-quencies (blue area) is shown for *Escherichia coli* sample. The genome size, heterozygote ratio and repetitive content of unprocessed short reads can be read.


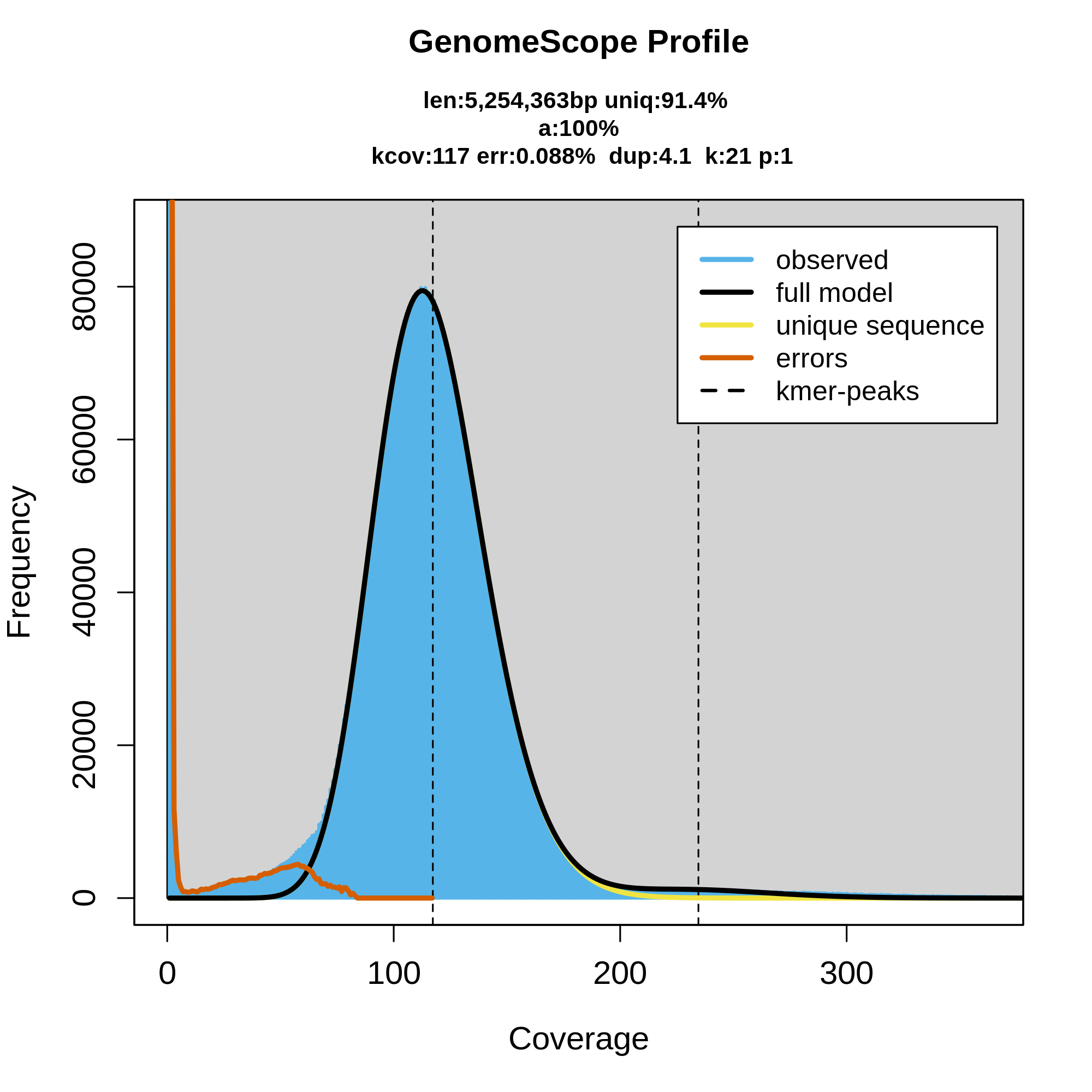
**Supplementary Figure 5** GenoScope profile of 1000× PSA sample. The fit of the model (black line) to the observed kmer fre-quencies (blue area) is shown for *Escherichia coli* sample. The genome size, heterozygote ratio and repetitive content of unprocessed short reads can be read.
